# Factors of migrant children’s participation in basic medical insurance in China: an empirical study based on DEMATEL-ISM method

**DOI:** 10.3389/fpubh.2024.1343860

**Published:** 2024-11-22

**Authors:** Hongyan Li, Chuanghao Yang, Zhiyang Liu, Zhihao Chen

**Affiliations:** School of Management, Shanghai University of Engineering Science, Shanghai, China

**Keywords:** basic medical insurance, migrant children, DEMATEL-ISM method, collaboration, factor hierarchy, China

## Abstract

**Background:**

Deepening medical insurance reform is pivotal in promoting fairness, inclusiveness, and sustainability within the system, particularly by enhancing coordination levels and strengthening the interconnection between medical insurance, healthcare, and pharmaceuticals. In China, 71.09 million migrant children, who make up 23.86% of the total child population, exhibit lower participation rates in medical insurance compared to other groups. The health status of children serves as a crucial barometer for the country’s socioeconomic standing and the quality of its medical services. Therefore, the inclusion of migrant children in basic medical insurance is essential for elevating their health standards and contributing to the accumulation of human capital.

**Method:**

This study employed the structural-dynamic-process-result framework derived from synergy theory. It identified 18 factors influencing the participation of migrant children in basic medical insurance. Utilizing the DEMATEL-ISM method, the research analyzed these factors, culminating in the development of a comprehensive five-level hierarchical structure model.

**Result:**

The research identified the medical insurance system’s arrangements as central to influencing the participation of migrant children in medical insurance. The household registration system emerged as a critical factor with significant mandatory and motivational impacts. The study categorized the 18 influencing factors into three tiers: superficial inducing factors, intermediate influencing factors, and deep-rooted causal factors. These tiers demonstrate a complex web of interdependencies and influences, highlighting that encouraging migrant children’s participation in medical insurance is a multifaceted systemic endeavor. This process necessitates collaborative efforts from families, schools, markets, society, and government bodies.

**Conclusion:**

To effectively foster the participation of migrant children in basic medical insurance, a strong focus on identifying and addressing core issues is required. This approach should be coupled with enhanced strategic planning and coordination skills to ensure that reforms and developmental strides are equitably beneficial. Recommendations include decentralizing children’s medical insurance from local constraints, refining insurance system design, elevating the level of medical insurance coordination, and boosting insurance participation efficiency. Additionally, fortifying the collaborative dynamics among healthcare, medical insurance, and pharmaceutical sectors is crucial in building a united front to support migrant children’s healthcare needs.

## 1 Introduction

The pursuit of health is a universal aspiration, and “universal health coverage” is a key goal endorsed by the international community. Basic medical insurance, as an institutional arrangement, plays a crucial role in dispersing disease risks, reducing economic burdens, and promoting better health outcomes. Achieving comprehensive coverage of this insurance is essential for realizing universal health coverage. In China, economic and social transformations have led to significant urban–rural and inter-regional population movements, resulting in a growing number of migrant children. According to the ‘Seventh Census’ and the ‘China Migrant Children Development Report 2021’, there are approximately 130 million children within the migrant population, with 71.09 million identified as migrant children, representing about 23.86% of China’s total child population. This substantial number highlights the significance of addressing the healthcare needs of this demographic. However, their participation in basic medical insurance lags behind other groups (1). With China’s demographic shift toward a lower birth rate and the implementation of supportive birth policies, children’s health levels have become a crucial indicator of the country’s socioeconomic and healthcare standards. Including migrant children in basic medical insurance is vital for their health protection. Despite China’s universal medical security system, which nominally covers urban and rural children (2), the localized management of basic medical insurance often hinders migrant children from accessing complete and effective coverage. This gap leads to the underutilization of medical services and compromises their health rights. Addressing this issue is urgent for several reasons. First, ensuring migrant children’s participation in basic medical insurance is a valuable investment in human capital, promoting their healthy development and contributing to the vision of a ‘healthy China’ and a talent-strong nation. Second, their inclusion can enhance the effectiveness of the insurance system and its overall coordination. Streamlining services like out-of-town medical settlements and transfer continuity is crucial for a fair, unified, and sustainable social security system. Lastly, enabling migrant children’s participation in basic medical insurance helps share the economic burden of medical care with their families, aligning with the goal of achieving a better quality of life for all, fostering social equity and justice, and supporting high-quality development and common prosperity.

## 2 Factors of migrant children’s participation in medical insurance

Research on the factors influencing participation in insurance has been a prominent topic both domestically and internationally. Scholars use both qualitative and quantitative methods to analyze the factors affecting migrant children’s participation in basic medical insurance. While these studies have produced substantial theoretical insights, they often fall short in exploring the depth and breadth of influencing factors, particularly overlooking the impact of institutional design and other systemic factors. For instance, Rational Choice Theory focuses on individual decision-making processes, but may not adequately account for systemic influences. Social Determinants Theory emphasizes broader social and economic factors but lacks a detailed analysis of structural and dynamic interactions. Institutional Theory examines the role of institutions and policies but might overlook the dynamic interplay between different subsystems.

Firstly, the current state of migrant children’s participation in basic medical insurance reveals two main issues. On one hand, their participation rates are generally low, with some central and western regions reporting rates below 20% (3). On the other hand, due to the household registration system and policy restrictions, migrant children often cannot access the same medical benefits as local children in urban areas (4). This disparity is compounded by a general lack of awareness about medical insurance among migrant families. Additionally, several families with migrant children demonstrate limited understanding of medical insurance, delaying or preventing their participation (1). This issue might be linked to factors such as the family’s educational background and inadequate dissemination of information about medical insurance programs (5).

Secondly, when examining the factors affecting migrant children’s participation in medical insurance, existing research spans various dimensions such as policy, economy, culture, and family. China has implemented several policy measures to address medical security for migrant children, including facilitating medical treatment in different locations, direct settlement of medical bills, and simplifying the reimbursement process for out-of-town treatments (6, 7). Despite these efforts, challenges persist in the practical implementation of these policies, such as inadequate information sharing, low reimbursement rates, and complex reimbursement procedures (8). Moreover, family economic status plays a significant role in the willingness and ability of migrant children to participate in medical insurance. Families with lower incomes often prioritize basic necessities over medical insurance, which can be financially burdensome (9). Financial constraints may also lead some families to delay or forgo medical treatment, causing children to miss timely healthcare (10, 11). Cultural factors, including parents’ education level and awareness of medical insurance, significantly influence migrant children’s participation in medical insurance (12, 13). Additionally, family stability and the parents’ understanding of medical insurance, as well as their focus on children’s health, are crucial factors. Migrant families often face instability and prolonged periods away from home, leading to less attention paid to children’s health (14). Furthermore, the pressures of work and life can make it challenging for parents to fully engage with their children’s medical insurance issues (15).

Thirdly, in the realm of research methods, scholars have employed a variety of approaches to study migrant children’s participation in basic medical insurance. These methods include literature analysis, empirical research, survey research, and policy analysis. Some scholars have conducted comprehensive reviews and analyses of both domestic and international research literature, focusing on factors influencing participation, current status, policy measures, and more (4, 16). Commonly used statistical tools in these studies include descriptive statistics, regression analysis, and the basic entry tree model method (1, 12, 17, 18). In addition to these, some researchers have also focused on case studies to gain a deeper understanding of migrant children’s experiences with medical insurance, extracting valuable insights and lessons (19, 20). Questionnaire surveys and interviews are another significant approach, helping to gather data on the attitudes, willingness, and challenges faced by migrant children and their families in participating in medical insurance (21–23). Additionally, some scholars have evaluated the effectiveness of current policies, assessing their impact on migrant children’s participation in medical insurance and offering recommendations for policy improvement (24, 25). However, empirical methods may be influenced by the subjective judgment of researchers, which to some extent limits their objectivity. Principal Components Analysis (PCA) may introduce a certain degree of ambiguity, making it difficult to accurately convey its practical significance (26). The SEM method is more flexible in modeling latent variables but requires larger sample sizes (27). ANP, on the other hand, is better suited for decision-making processes where multiple criteria and interdependencies require simultaneous consideration (28). While multivariate regression can identify the strength of relationships between variables, it does not inherently provide a hierarchical or network structure (29). Compared to the above methods, DEMATEL-ISM method can not only clarify key factors, but also sort out the logical relationships between factors. Therefore, this research adopts the DEMATEL-ISM method to clarify the internal connections and hierarchical structure among the factors.

Despite these efforts, there appears to be a lack of systematic analysis by practitioners and academic circles on the factors affecting migrant children’s participation in basic medical insurance. This study employs Haken’s synergetics theory, which provides a comprehensive framework for understanding complex systems through the lens of self-organization, feedback mechanisms, and nonlinear interactions. Synergetics theory is particularly advantageous for this study as it allows for the examination of both micro and macro-level influences and their interdependencies, making it well-suited for analyzing the multifaceted factors affecting migrant children’s participation in basic medical insurance. By using the DEMATEL-ISM method within this theoretical framework, this research aims to identify and clarify the hierarchical structure and interactions among these factors, offering a more holistic understanding and practical recommendations. This research not only aims to protect the rights and interests of migrant children in this arena but also seeks to achieve comprehensive coverage under the basic medical insurance system.

The study analyzed the factors affecting migrant children’s participation in basic medical insurance from the four dimensions of synergy theory: structural characteristics, dynamic characteristics, process characteristics, and result characteristics, explaining each factor as shown in Table 1.

**Table 1:** Factors influencing migrant children’s participation in medical insurance.

	Characteristics	Serial number	Influencing factors	Definition
Factors influencing migrant children’s participation in basic medical insurance	Structural characteristics	S_1_	Household registration system	The floating population residence permit system allows holders of residence permits to enjoy the treatment of local residents.
		S_2_	The education policy of the place of immigration	The admission threshold for migrant children, whether they have a residence permit, and how many points they are worth when applying for points for migrant children.
		S_3_	Family’s financial situation	The level of disposable income of family members and the status of family property.
		S_4_	Parents’ engage in career stability	Have a fixed job for a certain period of time and will not leave or be fired easily.
		S_5_	Sense of belonging to city	An important indicator of social integration, “localized” awareness can increase the willingness of migrants to participate in insurance to a certain extent.
	Dynamic characteristics	S_6_	Herd mentality	The tendency of families to collectively follow their community’s behavior and decisions when enrolling in insurance programs without centralized direction.
		S_7_	Family risk attitude	Family attitude toward health risks for migrant children.
		S_8_	Insurance policy publicity	The publicity and interpretation of the basic medical insurance policy by the government, society and schools.
		S_9_	Supplementary medical insurance	Generally refers to commercial insurance, the purchase of hospitalization insurance protection products.
	Process characteristics	S_10_	Medical insurance system arrangements	The place of inflow shall implement specific policies and business operations to prevent and treat residents’ diseases in accordance with insurance principles.
		S_11_	Medical security benefit level	The level of personal security benefits at the micro level. The level of medical expense reimbursement in the place of influx.
		S_12_	Insurance fee	The payment standards for basic medical insurance shall apply to the place of inflow.
		S_13_	Medical insurance deductible	The standard amount of hospitalization medical expenses borne by migrant children in the place of influx.
		S_14_	Medical insurance reimbursement ratio	The reimbursement ratio of medical expenses in the place of influx meets the prescribed range.
		S_15_	Medical insurance handling service level and efficiency	The service level and processing efficiency provided by the medical insurance institutions in the place of influx when processing medical insurance business.
	Result characteristics	S_16_	Health level of migrant children	The comprehensive health status of migrant children in many aspects such as physical, psychological and social adaptation.
		S_17_	Children’s medication reserve	In the case of children’s drug treatment, medical institutions, pharmacies, etc., have delayed the stocking of medicines used by children.
		S_18_	Access to medical services	The degree of barriers in time, space, information, cost, etc., between the medical resources of the place of influx and migrant children.

## 3 Theoretical framework

### 3.1 Synergy theory mechanism

In the early 1970s, German physicist Hermann Haken pioneered the field of synergetics, which has since become a pivotal branch of systems science. Synergetics examines systems in nature and society, positing that all systems are divisible into various subsystems. These subsystems interact within a complex composite system, with synergetic interactions governed by universal principles, irrespective of the subsystems’ nature (30). Synergy theory believes that there is a mutual influence and cooperation relationship between various systems in the entire environment, so it is used to describe self-organization phenomena and collaborative behaviors in complex systems (31). Synergy theory emphasizes the following points.

First, the phenomenon of self-organization. Synergy theory that composite systems, under certain conditions, will spontaneously form ordered structures or patterns. This phenomenon of self-organization is propelled by the interactions and feedback mechanisms within the system. Second, the aggregation behavior. Within a composite system, subsystems, individuals, or elements often spontaneously converge into groups, forming larger-scale structures. This behavior is governed by basic interaction rules such as attraction, repulsion, and synchronization. Third, feedback mechanisms. Various feedback mechanisms, including both positive and delayed feedback, are present in composite systems. These mechanisms are crucial in enhancing the system’s stability and adaptability, leading to more sophisticated collaborative behavior. Forth, resonance. Elements within a composite system interact, creating resonance phenomena. This resonance amplifies the system’s sensitivity and responsiveness, enabling more efficient cooperative behavior. Fifth, nonlinearity. Interactions within composite systems are often nonlinear, meaning they do not follow simple linear relationships. This nonlinearity can result in diverse behaviors and phenomena, including chaos, self-similarity, and fractal patterns. These points together constitute the important ideas of synergy theory, which can be used to describe and explain various natural and social phenomena.

### 3.2 Characteristic analysis framework of synergy theory

Haken’s synergy theory offers an insightful framework for delving into the complexities of systems and the effects of synergistic interactions. This study distills the theory into a comprehensive characteristic analysis framework, encapsulating structural, dynamic, process, and result. Within the structural dimension, elements such as organizational design, role distribution, and the sharing of information and knowledge are pivotal. The dynamic dimension highlights the importance of shared goals, interactive communication, and motivational incentives. In terms of process characteristics, the focus is on collaborative procedures, decision-making, problem-solving, coordination, and technological backing. Lastly, the results dimension encompasses aspects like collaborative outcomes, participant contentment, and contributions to learning, development, innovation, and adaptability. This multifaceted approach provides a detailed exploration of collaborative behavior across four key dimensions.

#### 3.2.1 Structural characteristics

Structural characteristics are an important component of collaborative behavior and involve the social and organizational structure between subsystems. In the structural characteristics dimension, the following two aspects are included. First, organizational structure. Collaborative behavior can occur in different organizational structures, such as hierarchical, networked or decentralized organizational structures. Different organizational structures will affect information flow and decision-making. These include factors such as process and power distribution. Second, role and responsibility allocation. Subsystems play different roles and share different responsibilities in collaborative behavior. The clarity of roles and responsibilities is crucial to the effectiveness and efficiency of collaborative behavior.

Reflecting these structural characteristics, the subsequent examination focuses on the social and familial contexts influencing migrant children’s participation in basic medical insurance. Influenced by the household registration system (S_1_) and the education policy of the place of immigration (S_2_), the eligibility of migrant children for local basic medical insurance is determined. Additionally, the family’s financial situation (S_3_) gives families different financial strengths, which in turn influences parents’ ability to purchase medical insurance for their children. In addition, parents’ engage in career stability (S_4_) also plays a role, affecting both the family’s economic situation and their inclination to invest in medical insurance. Finally, the sense of belonging to the city (S_5_) can impact migrant families’ desire to integrate into the local community and participate in its healthcare system.

#### 3.2.2 Dynamic characteristics

Dynamic characteristics focus on the motivations and motivating factors of synergistic phenomena. In the dynamic characteristics dimension, the following aspects should be considered. First, information and knowledge sharing. Collaborative behavior requires information and knowledge sharing between subsystems. Effective information and knowledge sharing can promote synergy between subsystems. Second, goal sharing. The core of collaborative behavior is sharing a common goal. Shared goals can stimulate motivation among systems and promote cooperation and coordinated action. Third, interaction and communication. Collaborative behavior requires effective interaction and communication. Effective interaction and communication can build trust, understanding and consensus, and promote cooperation and coordination. Forth, power incentive mechanism. Collaborative behavior requires appropriate incentive mechanisms to stimulate enthusiasm and effort. Incentives can include financial rewards, recognition, and a sense of accomplishment.

Considering these dynamic characteristics, several factors are identified that affect migrant children’s participation in basic medical insurance. Migrant children’s decisions may be swayed by a herd mentality (S_6_), reflecting the influence of their immediate social environment. The family risk attitude (S_7_) also shapes their willingness to purchase medical insurance for their children and their cost investment in resisting risks. Furthermore, insurance policy publicity (S_8_) through government, societal, and educational channels can modify families’ and migrant children’s perceptions and eagerness to engage with medical insurance. Lastly, families’ decisions to opt for supplementary medical insurance (S_9_) can influence their reliance on basic medical insurance.

#### 3.2.3 Process characteristics

Process characteristics focus on the specific operations and implementation of collaborative behavior. In the process characteristic dimension, this includes the following aspects. First, collaborative process. Collaborative behavior requires clear collaborative processes and operational steps. The design and implementation of collaborative processes can improve collaborative efficiency and effectiveness. Second, decision-making and problem-solving. The decision-making and problem-solving processes in collaborative behavior is crucial to collaborative results. Reasonable decision-making and problem-solving mechanisms can reduce conflicts and obstacles and improve collaborative effectiveness. Third, technical support. Collaborative behavior can be supported with the help of technical tools and platforms. Technical support can provide functions such as information sharing, collaboration tools, and collaborative management, thereby improving collaborative efficiency.

In alignment with these process characteristics, several specific operations and implementations influence participation in basic medical insurance. Initially, regional variations in the medical insurance system (S_10_) can affect the ease of including migrant children in these programs. The level of medical security benefits (S_11_) influences family satisfaction and their continued participation in the insurance. Additionally, the level of insurance fee (S_12_) plays a role in family decisions regarding medical insurance purchases. The setting of the medical insurance deductible (S_13_) and the level of medical insurance reimbursement ratio (S_14_) further impact families’ dependency on and satisfaction with medical insurance. Finally, the service level and efficiency of medical insurance handling (S_15_) can affect families’ recognition of medical insurance and their willingness to continue participating in insurance.

#### 3.2.4 Result characteristics

Result characteristics focus on the actual effects and impacts of collaborative actions. In terms of the outcome characteristic dimension, the following aspects can be considered. First, collaborative performance. The performance of collaborative behavior is an important indicator to measure the synergistic effect. Collaborative performance includes aspects such as outcome quality, efficiency, innovation, and satisfaction. Second, learning and development. Collaborative behavior can promote learning and development of each subsystem. Through collaborative behavior, individual subsystems gain new skills and experience. Third, innovation and adaptability. Collaborative behavior can promote the improvement of innovation and adaptability. Through multi-party cooperation and knowledge sharing, collaborative behavior can stimulate innovative thinking and the ability to adapt to changes.

**Figure 1:**
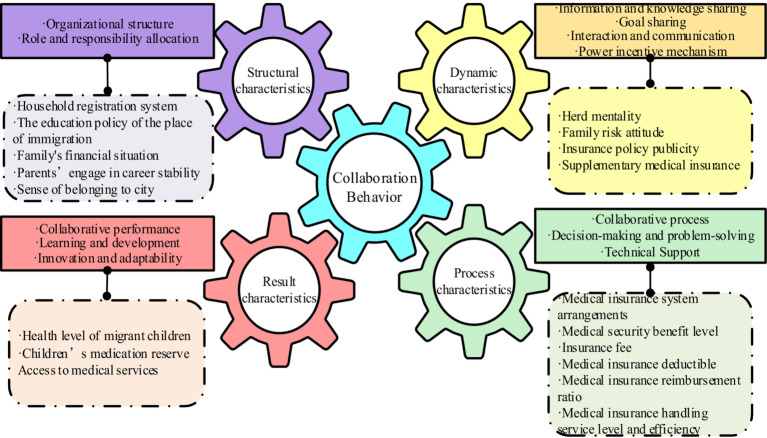
Analysis framework of synergy theory characteristics.

In relation to these result characteristics, several tangible outcomes and effects of participation in basic medical insurance are considered. First, after participating in medical insurance, whether the health level (S_16_) of migrant children has been improved will affect the family’s recognition of medical insurance. In addition, the adequacy of children’s medical reserves (S_17_) also influences the degree to which families rely on medical insurance when facing medical expenses. Finally, the access to medical services (S_18_) shapes family decisions in selecting medical insurance and their willingness to participate in such schemes (Figure 1).

In summary, the comprehensive analysis of the structural-dynamic-process-result characteristic framework based on synergy theory can comprehensively analyze the characteristics and influencing factors of collaborative behavior, and provide a unique research perspective for studying the many influencing factors of synergistic phenomena, offering theoretical guidance and practical application as a reference. This analytical framework is versatile and can be applied to a variety of collaborative environments and fields. This article will draw on the perspective of the characteristics of synergy theory, review the institutional change path of Chinese migrant children’s participation in basic medical insurance since the founding of the People’s Republic of China, and combine this with the macro background of China’s economic and social development and the development trend of medical security to provide a guide for the healthy development and development of Chinese children in the new era. Institutional innovation offers strategic optimization.

## 4 Methods

### 4.1 DEMATEL-ISM analysis method

**Figure 2:**
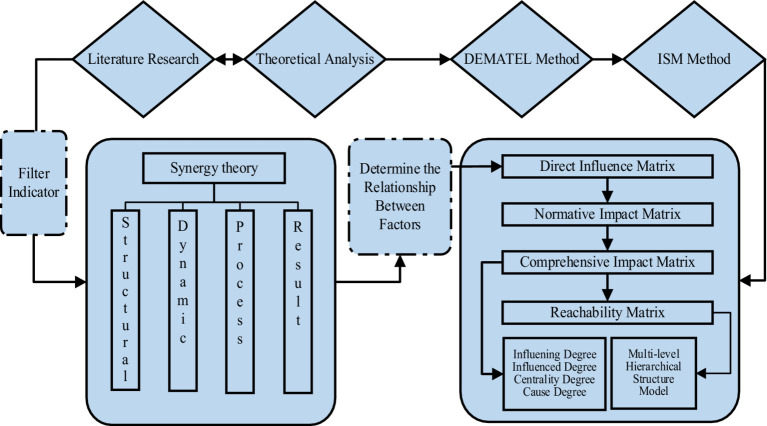
Idea diagram for DEMATEL-ISM analysis method construction.

DEMATEL method, the decision-making trial and evaluation laboratory method, is a complex system factor for analysis method jointly proposed by Fontela et al. This method uses graph theory and matrices to make qualitative judgments on factors in complex systems, and then calculates the degree of influence, influence, centrality, and cause of each factor, in order to clarify the interactions between factors in complex systems, identify the key elements of the system, and simplify the system structure (32). ISM method, the Interpretive Structural Model method, is an approach proposed by American systems engineering theorist Warfield to explain the structural relationships of system influencing factors. This method divides a complex system into multiple subsystems, uses matrices to process the relationships between factors, and ultimately forms a multi-layer hierarchical structure model. This transforms fuzzy and complex relationships into intuitive and clear structural relationships, making it suitable for analyzing and understanding complex systems with multiple influencing factors (33). The DEMATEL method focuses on micro-level analysis, and the ISM method is biased toward macro-level analysis (34). Integrating the DEMATEL and ISM methods to study the factors affecting migrant children’s participation in basic medical insurance can complement each other’s advantages, improve calculation efficiency, identify key elements in the system and their impact, and build a hierarchical structure of system elements. The specific construction concept is shown in Figure 2.

#### 4.1.1 Establishing the initial direct influence matrix

The expert scoring method is utilized to compare the impact of *S_i_* on *S_j_*. Since a factor has no influence on itself, the diagonal values of the direct influence matrix are set to zero. After this comparison, the direct impact matrix *A* can be obtained.


(1) $${S_{i}}_{j}=\{\begin{array}{c} 0,0\leq \alpha <0.5\\ 1,0.5\leq \alpha <1.5\\ 2,1.5\leq \alpha <2.5\\ 3,2.5\leq \alpha <3.5\\ 4,3.5\leq \alpha <4.0 \end{array}$$

(2) $$A=\left[\begin{array}{c} 0\;S_{12}\;\cdots \;S_{1j}\\ \begin{array}{cccc} S_{21} & 0 & \cdots & S_{2j} \end{array}\\ \begin{array}{cc} \begin{array}{cc} \begin{array}{cc} \cdots & \cdots \end{array} & \cdots \end{array} & \cdots \end{array}\\ \begin{array}{cccc} S_{i1} & S_{i2} & \cdots & 0 \end{array} \end{array}\right]$$

The factor *S_ij_* in the formula (*i* = 1, 2,…, *i*; *j* = 1, 2,…, *i* ≠ *j* represents the direct influence of factor *S_i_* on *S_j_*; if *i* = *j*, then *S_ij_* = 0).

#### 4.1.2 Normalizing the direct influence matrix

By normalizing the original relationship matrix, the normative direct influence matrix is obtained. This paper employs the row sum maximum method for normalization. This involves summing each row of matrix *A*, determining the maximum value among these sums, normalizing the factors in matrix *A* accordingly, and thereby obtaining the normative influence matrix *B*.


(3) $$B=\frac{S_{ij}}{\max\left(\sum_{j=1}^{n}S_{ij}\right)}$$

#### 4.1.3 Comprehensive impact matrix calculation

The comprehensive impact matrix represents the combined effects of direct and indirect impacts among system factors. After the normative influence matrix has been multiplied by itself, all values of the matrix will approach 0, that is $\lim_{n\to \infty }B^{k}$=0. Therefore, the comprehensive influence matrix *T* is obtained through the following formula.


(4) $$T=\left(B+B^{2}+\ldots +B^{k}\right)=\sum_{k=1}^{\infty }B^{k}=B{\left(I-B\right)}^{-1}$$

In Equation 4, *I* is the identity matrix.

#### 4.1.4 Assessing degrees of influence and centrality

The influencing degree refers to the sum of the values of each row in *T*, indicating the comprehensive influence of the corresponding elements of each row on all other elements, denoted as *D_i_*.


(5) $$D_{i}=\sum_{j=1}^{n}{S_{i}}_{j},\left(i=1,2,\ldots ,n\right)$$

The influenced degree refers to the sum of the values of each column in *T*, representing the comprehensive influence of the corresponding elements in each column on other elements, denoted as *C_i_*.


(6) $$C_{i}=\sum_{j=1}^{n}{S_{i}}_{j},\left(i=1,2,\ldots ,n\right)$$

Centrality degree represents the position and significance of a factor within the evaluation system. The sum of the influencing degree and the influenced degree of element i is the centrality of the element, denoted as *M_i_*, then,


(7) $$M_{i}=D_{i}+C_{i}$$

The cause degree is obtained by subtracting the influencing degree and the influenced degree of element *i*, denoted as *R_i_*, then,


(8) $$R_{i}=D_{i}-C_{i}$$

If the cause degree is greater than 0, it means that the element has a great influence on other elements and is called a cause element; otherwise, it is called a result element.

#### 4.1.5 Visualization of cause and effect relationships

Using the centrality *M_i_* as the abscissa and the cause *R_i_* as the ordinate, a causal relationship diagram is drawn to simplify the causal relationships.

#### 4.1.6 Computing the accessibility matrix

To calculate the accessibility matrix, the identity matrix *I* is introduced based on the comprehensive influence matrix *T* to construct the overall influence matrix *E*.


(9) $$E=T+I={\left[{e_{i}}_{j}\right]}_{n\times n}$$

A threshold *λ* is introduced to eliminate less influential relationships between factors, facilitating the division of hierarchical structures (35). Summarizing the current research by scholars, there are three main methods for determining the threshold *λ*. First, determine the threshold *λ* based on the empirical knowledge of experts and decision-makers; Second, calculate the mean value of the elements in the comprehensive impact matrix A to determine the threshold *λ*; Third, methods based on statistical distribution to determine the threshold *λ* (36). Given that the first method relies heavily on the subjective judgment of experts, the third method is an extension of the second method, incorporates the inherent laws of data distribution. Therefore, this paper determines the value of *λ* using the mean *ɑ* and standard deviation *θ* of all elements of the comprehensive influence matrix.


(10) λ=α+θ

By applying a threshold *λ* to process elements in the matrix *E*, the reachable matrix *F* is obtained.


(11) $$F={\left[{f_{i}}_{j}\right]}_{n\times n},f_{ij}=\{\begin{array}{c} 1,{e_{i}}_{j}\geq \lambda \left(i,j=1,2,\ldots n\right)\\ 0,{e_{i}}_{j}<\lambda \left(i,j=1,2,\ldots n\right) \end{array}$$

#### 4.1.7 Hierarchical structuring and factor levels

The accessibility level *R(S_i_) =* {*S_i_|F_ij_* = 1} is obtained through the factors corresponding to the columns with a value of 1 in the *i*-th row of the accessibility matrix *F*, which represents the set of all factors that can be reached starting from the factor *S_i_*. The factor corresponding to the column with a value of 1 on the *i*-th column of the accessibility matrix *F* finds the advance level *S(S_i_)* = {*S_i_|F_ij_* = 1}, which represents the set of all factors that can reach the factor S_i_; if *R(S_i_)* and *S(S_i_)* satisfy *R(S_i_)*∩*S(S_i_)* = *R(S_i_)*, it means that the corresponding factor *S_i_* in *R(S_i_)* can all find its antecedent in *S(S_i_)*. This factor is called located in high-level factors, then delete the corresponding rows and columns from the accessibility matrix, and then extract the highest-level factors from the remaining matrix, repeating this process until all rows and columns are deleted.

#### 4.1.8 Building the hierarchical structure model

According to the order in which factors are eliminated, a multi-level hierarchical structure model between system elements is drawn.

## 5 Results

### 5.1 DEMATEL-ISM model construction

This study distributed a survey to 10 experts familiar with children’s participation in basic medical insurance (including 3 from the Medical Insurance Bureau, 2 from the Civil Affairs Department, 2 from the health system, 2 from relevant research universities, and 1 from a commercial insurance company). The questionnaire rated the intensity of interaction between influencing factors on a scale from no impact (0 points), small impact (1 point), average impact (2 points), large impact (3 points), and very large impact (4 points). These five levels are assigned values to obtain 10 sets of influence matrices. To ensure internal consistency of the questionnaire data, Cronbach’s *α* coefficient test was conducted using SPSS. The *α* coefficient was 0.969, indicating good reliability as it was greater than 0.80. Then the average method is used to combine the scoring results of 10 experts and calculate the average value to eliminate the subjective differences among the experts. Ultimately, the initial direct influence matrix *A* is obtained according to Equation 1, as shown in Table 2.

**Table 2:** Initial direct influence matrix A.

Influencing factors	S_1_	S_2_	S_3_	S_4_	S_5_	S_6_	S_7_	S_8_	S_9_	S_10_	S_11_	S_12_	S_13_	S_14_	S_15_	S_16_	S_17_	S_18_
S_1_	0	4	1	1	3	0	0	1	0	4	4	4	4	4	4	1	4	4
S_2_	1	0	2	3	3	1	1	2	0	3	0	0	0	0	0	2	3	3
S_3_	0	0	0	3	3	2	4	0	4	0	0	0	0	0	0	3	0	0
S_4_	0	0	3	0	4	0	3	0	3	0	0	0	0	0	0	2	0	1
S_5_	0	0	3	4	0	2	2	1	2	0	0	0	0	0	0	2	1	1
S_6_	0	0	0	0	0	0	0	2	1	0	0	0	0	0	0	0	0	0
S_7_	0	0	2	1	2	1	0	2	4	0	0	0	0	0	0	2	0	0
S_8_	0	1	0	0	1	3	3	0	2	3	1	1	1	1	1	2	2	2
S_9_	0	0	0	0	0	1	2	2	0	0	0	0	0	0	0	3	0	0
S_10_	0	1	1	1	3	2	2	3	2	0	4	4	4	4	4	3	2	2
S_11_	0	0	3	1	2	1	1	1	1	3	0	1	1	3	1	2	1	2
S_12_	0	0	3	1	2	1	1	1	1	3	3	0	2	2	1	1	1	2
S_13_	0	0	3	1	2	1	1	1	1	3	3	1	0	2	1	1	1	2
S_14_	0	0	3	1	2	1	1	1	1	3	4	1	1	0	1	2	1	2
S_15_	0	0	3	1	3	1	2	3	1	3	1	1	1	1	0	2	1	2
S_16_	0	0	3	1	3	2	3	2	3	1	1	1	1	1	1	0	2	2
S_17_	0	0	2	2	4	1	3	1	3	3	1	1	1	1	1	3	0	3
S_18_	0	0	2	2	4	1	3	2	3	3	1	1	1	1	1	4	2	0

Using the row sum maximum method to normalize the table matrix, the normative influence matrix B can be obtained according to Equation 2, and the comprehensive influence matrix T can be obtained by calculating the normative influence matrix according to Equation 3, as shown in Table 3.

**Table 3:** Comprehensive impact matrix T.

Influencing factors	S_1_	S_2_	S_3_	S_4_	S_5_	S_6_	S_7_	S_8_	S_9_	S_10_	S_11_	S_12_	S_13_	S_14_	S_15_	S_16_	S_17_	S_18_
S_1_	0.00	0.10	0.13	0.10	0.19	0.07	0.10	0.10	0.10	0.18	0.16	0.14	0.14	0.15	0.14	0.13	0.15	0.17
S_2_	0.02	0.01	0.10	0.11	0.13	0.06	0.09	0.08	0.07	0.10	0.03	0.02	0.02	0.03	0.02	0.11	0.10	0.10
S_3_	0.00	0.00	0.03	0.09	0.10	0.07	0.12	0.02	0.13	0.01	0.01	0.00	0.01	0.01	0.00	0.10	0.01	0.01
S_4_	0.00	0.00	0.09	0.03	0.12	0.02	0.10	0.02	0.11	0.01	0.01	0.00	0.01	0.01	0.00	0.08	0.01	0.03
S_5_	0.00	0.00	0.10	0.11	0.04	0.07	0.08	0.04	0.09	0.01	0.01	0.01	0.01	0.01	0.01	0.08	0.03	0.04
S_6_	0.00	0.00	0.00	0.00	0.00	0.01	0.01	0.05	0.03	0.01	0.00	0.00	0.00	0.00	0.00	0.01	0.00	0.00
S_7_	0.00	0.00	0.06	0.04	0.07	0.04	0.03	0.06	0.12	0.01	0.01	0.01	0.01	0.01	0.01	0.07	0.01	0.01
S_8_	0.00	0.03	0.05	0.03	0.08	0.10	0.12	0.04	0.10	0.10	0.05	0.04	0.05	0.05	0.04	0.10	0.07	0.08
S_9_	0.00	0.00	0.01	0.01	0.02	0.04	0.06	0.06	0.02	0.01	0.01	0.01	0.01	0.01	0.01	0.08	0.01	0.01
S_10_	0.00	0.03	0.12	0.08	0.17	0.11	0.13	0.13	0.14	0.07	0.14	0.12	0.13	0.14	0.12	0.16	0.09	0.11
S_11_	0.00	0.00	0.12	0.06	0.11	0.06	0.08	0.06	0.08	0.10	0.03	0.05	0.05	0.09	0.05	0.10	0.05	0.08
S_12_	0.00	0.00	0.12	0.06	0.11	0.06	0.08	0.06	0.08	0.11	0.10	0.02	0.07	0.08	0.05	0.08	0.05	0.08
S_13_	0.00	0.00	0.12	0.06	0.11	0.06	0.08	0.06	0.08	0.11	0.10	0.05	0.02	0.07	0.05	0.08	0.05	0.08
S_14_	0.00	0.00	0.12	0.06	0.11	0.06	0.08	0.06	0.08	0.11	0.12	0.05	0.05	0.03	0.05	0.11	0.05	0.08
S_15_	0.00	0.01	0.12	0.06	0.13	0.07	0.11	0.11	0.09	0.10	0.05	0.05	0.05	0.05	0.02	0.11	0.05	0.08
S_16_	0.00	0.00	0.12	0.06	0.12	0.08	0.12	0.08	0.13	0.06	0.05	0.04	0.04	0.04	0.04	0.06	0.07	0.07
S_17_	0.00	0.00	0.11	0.09	0.16	0.07	0.14	0.07	0.14	0.10	0.05	0.05	0.05	0.05	0.05	0.14	0.03	0.10
S_18_	0.00	0.01	0.11	0.09	0.16	0.07	0.14	0.09	0.14	0.11	0.05	0.05	0.05	0.05	0.05	0.16	0.08	0.04

Calculate Table 3 according to Equations 4–7, and can get the influencing degree, influenced degree, centrality degree and cause degree of each influencing factor, see Table 4.

**Table 4:** The influence degree, influenced degree, centrality degree and cause degree of each influencing factor.

Influencing factors	Influencing degree		Influenced degree		Centrality degree		Cause degree	
	DI	Rank/Position	*CI*	Rank/Position	*M*	Rank/Position	*R*	Rank/Position
S_1_	2.249	1	0.028	18	2.277	7	2.221	1
S_2_	1.195	8	0.201	17	1.396	17	0.995	2
S_3_	0.709	14	1.613	5	2.322	6	−0.904	14
S_4_	0.639	15	1.136	9	1.776	16	−0.497	12
S_5_	0.722	11	1.898	1	2.620	3	−1.176	17
S_6_	0.130	18	1.101	10	1.232	18	−0.971	15
S_7_	0.550	16	1.666	4	2.216	9	−1.116	16
S_8_	1.129	10	1.216	7	2.345	5	−0.086	11
S_9_	0.349	17	1.718	3	2.067	12	−1.368	18
S_10_	1.983	2	1.305	6	3.288	1	0.678	3
S_11_	1.166	12	0.970	11	2.137	10	0.196	10
S_12_	1.214	7	0.689	15	1.903	14	0.526	5
S_13_	1.165	13	0.727	14	1.892	15	0.438	7
S_14_	1.213	6	0.856	13	2.069	11	0.358	8
S_15_	1.237	5	0.689	15	1.926	13	0.548	4
S_16_	1.180	9	1.745	2	2.924	2	−0.565	13
S_17_	1.379	4	0.894	12	2.273	8	0.485	6
S_18_	1.421	3	1.182	8	2.603	4	0.239	9

**Figure 3:**
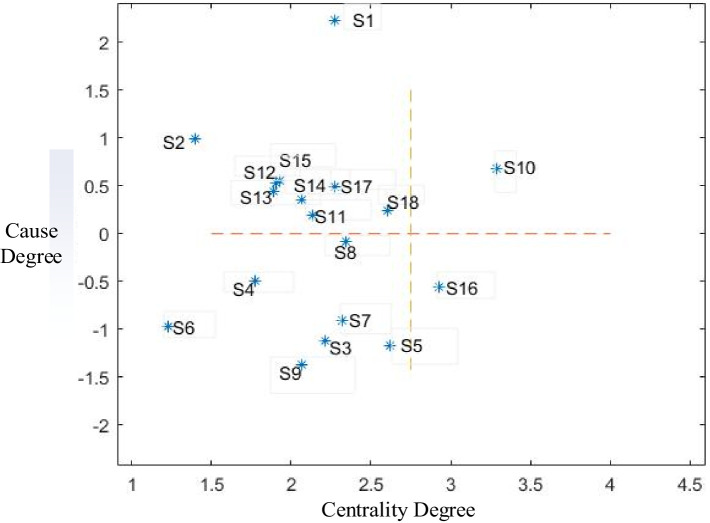
Factors affecting children’s participation in basic medical insurance—result chart.

According to the centrality and cause degree values of each forming factor calculated in Table 4, Matlab software was used to draw a cause-result diagram of factors affecting children’s participation in basic medical insurance, as shown in Figure 3.

The overall impact matrix is obtained by adding the comprehensive impact matrix from Table 3 to the unit matrix *I*. To derive the accessibility matrix, a threshold *λ* is introduced to eliminate relationships with minimal influence. The threshold λ is set at 0.107 based on the mean *α* and standard deviation *θ* of all elements in the comprehensive influence matrix. Based on the overall influence matrix result and Equations 8–11, the accessibility matrix of factors affecting migrant children’s participation in basic medical insurance is presented in Table 5.

**Table 5:** Reachable matrix F.

Influencing factors	S_1_	S_2_	S_3_	S_4_	S_5_	S_6_	S_7_	S_8_	S_9_	S_10_	S_12_	S_13_	S_14_	S_15_	S_16_	S_17_	S_18_
S_1_	1	0	1	1	1	0	1	1	1	1	1	1	1	1	1	1	1
S_2_	0	1	0	1	1	0	0	0	0	0	0	0	0	0	0	0	0
S_3_	0	0	1	0	0	0	1	0	1	0	0	0	0	0	0	0	0
S_4_	0	0	0	1	1	0	0	0	0	0	0	0	0	0	0	0	0
S_5_	0	0	0	1	1	0	0	0	0	0	0	0	0	0	0	0	0
S_6_	0	0	0	0	0	1	0	0	0	0	0	0	0	0	0	0	0
S_7_	0	0	0	0	0	0	1	0	1	0	0	0	0	0	0	0	0
S_8_	0	0	0	0	0	1	1	1	1	0	0	0	0	0	0	0	0
S_9_	0	0	0	0	0	0	1	0	1	0	0	0	0	0	0	0	0
S_10_	0	0	1	1	1	0	1	1	1	1	1	1	1	1	1	0	0
S_11_	0	0	1	0	0	0	1	0	1	0	0	0	1	0	0	0	0
S_12_	0	0	1	0	0	0	1	0	1	0	1	0	0	0	0	0	0
S_13_	0	0	1	0	0	0	1	0	1	0	0	1	0	0	0	0	0
S_14_	0	0	1	0	0	0	1	0	1	0	0	0	1	0	0	0	0
S_15_	0	1	1	1	1	0	1	1	1	0	0	0	0	1	0	0	0
S_16_	0	0	1	1	1	0	1	0	1	0	0	0	0	0	1	0	0
S_17_	0	0	1	1	1	0	1	0	1	0	0	0	0	0	1	1	0
S_18_	0	0	1	1	1	0	1	0	1	0	0	0	0	0	1	0	1

**Figure 4:**
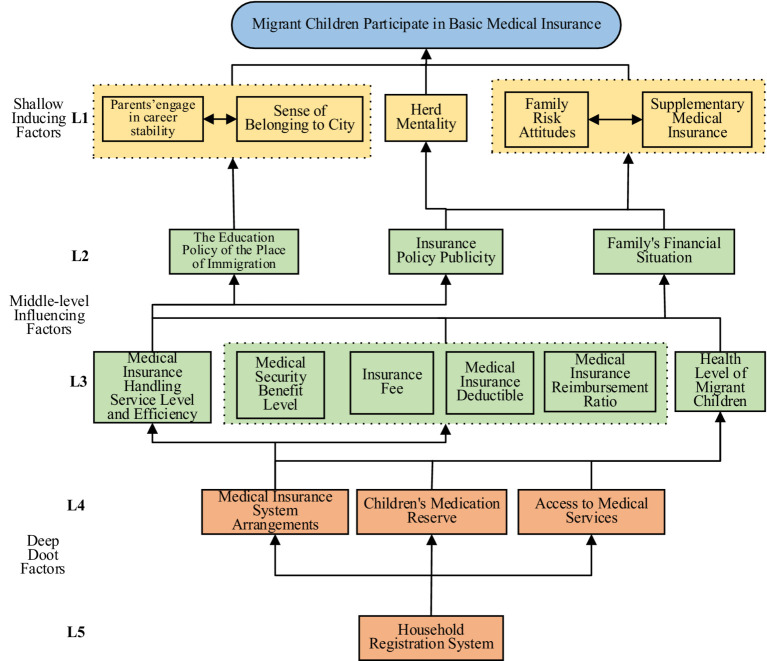
Hierarchical structure model diagram of factors influencing children’s participation in basic medical insurance.

From Table 5, the reachable set *R*(*S_i_*) and the antecedent set *A*(*S_i_*) for factors influencing migrant children’s participation in basic medical insurance are determined. It has been verified that when *i* = 4, 5, 6, 7, 9, *R*(*S_i_*)∩*A*(*S_i_*) = *R*(*S_i_*), S_1_, S_4_, S_6_, S_8_ and S_9_ are the first-layer formation factors; it’s crossed out the rows and columns corresponding to these factors in the matrix and repeat the above steps to get the first layer formation factors. The rows and columns corresponding to these factors are crossed out in the matrix, and the process is repeated to identify the second-layer formation factors S_2_, S_3_, and S_8_. Similarly, the third-layer formation factors S_11_, S_12_, S_13_, S_14_ and S_15_, S_16_ can be obtained; the fourth-layer formation factors S_10_, S_17_ and S_18_. The fifth-layer formation factor is S_1_. Consequently, the factors influencing migrant children’s participation in basic medical insurance are categorized into five levels, as illustrated in Figure 4.

### 5.2 Result analysis

#### 5.2.1 Analysis influencing degree, influenced degree, centrality degree and cause degree

First, by analyzing the calculated influence and degree of influence of factors affecting migrant children’s participation in basic medical insurance (Table 4), it can be seen that the top five influences are: household registration system (S_1_), medical insurance system arrangements (S_10_), access to medical services (S_18_), children’s medication reserve (S_17_), and medical insurance handling service level and efficiency (S_15_). These five factors have a significant influence and are the main influencing factors for migrant children to participate in basic medical insurance. The top five most influenced factors are: medical security benefit level (S_11_), health level of migrant children (S_16_), supplementary medical insurance (S_9_), family risk attitude (S_7_), and family’s financial situation (S_3_). These five factors are highly reactive to external influences and significantly affect how migrant children engage with basic medical insurance.

Next, analyze the centrality degree (Table 4) and find that the top five are: medical insurance system arrangements (S_10_), health level of migrant children (S_16_), sense of belonging to city (S_5_), access to medical services (S_18_), and insurance policy publicity (S_8_). This analysis highlights the medical insurance system arrangements (S_10_) occupy a central position and have a decisive influence on the impact of migrant children’s participation in medical insurance. The centrality degree of health level of migrant children (S_16_) ranks second in the entire system, indicating that it is a pivotal factor. The sense of belonging to city (S_5_), access to medical services (S_18_), and insurance policy publicity (S_8_) have high centrality degree, suggesting that they are important points to promote migrant children to participate in basic medical insurance. Conversely, the centrality degree rankings of the education policy of the place of immigration (S_2_) and herd mentality (S_6_) are low, indicating that these two factors are lesser importance. Other factors play a more important role and status in the entire system.

Finally, examining the cause degree, the top five are: household registration system (S_1_), the education policy of the place of immigration (S_2_), medical insurance system arrangements (S_10_), medical insurance handling service level and efficiency (S_15_), insurance fee (S_12_). Among the factors that affect migrant children’s participation in basic medical insurance, the household registration system (S_1_) is the causal factor and has the largest causality and the greatest impact on other factors. Therefore, the household registration system (S_1_) must be improved as a priority. If the reason degree is greater than 0, such as the education policy (S_2_) and medical insurance system arrangement (S_10_) of the place of immigration, it is also the reason factor that must be focused on. In addition, like supplementary medical insurance (S_9_), sense of belonging to the city (S_5_), family risk attitude (S_7_), herd mentality (S_6_), family’s financial situation (S_3_), health level of migrant children (S_16_), parents’ engage in career stability (S_4_) and insurance policy publicity (S_8_) are all less than zero. These factors are the most sensitive to other factors. Therefore, we must pay close attention to and control these factors and actively explore the factors that affect migrant children’s participation in basic medical insurance. In addition, the cause degree of influencing factors in the system includes both cause factors greater than zero and result factors less than zero, indicating that the influencing factors have important influences and constraints, and further analysis needs to be done with the help of hierarchical structure model.

#### 5.2.2 Hierarchical structure model analysis

The ISM model categorizes the factors influencing migrant children’s engagement with basic medical insurance into a hierarchical structure with three tiers and five levels (Figure 4): shallow inducing factors (first level); middle level inducing factors (second level and third level); deep root factors (fourth level and fifth level). Moreover, the factors influencing migrant children’s participation in basic medical insurance show relatively complex relationships such as same-level or cross-level interactions.

As the first-level factors that affect migrant children’s participation in basic medical insurance, superficial inducing factors will have the most direct impact. Mainly include parents’ engage in career stability (S_4_), sense of belonging to the city (S_5_), herd mentality (S_6_), family risk attitude (S_7_), and supplementary medical insurance (S_9_). These factors reflect the structural and dynamic characteristics of factors affecting migrant children’s participation in basic medical insurance, and there is mutual influence between factors.

The middle-level factors, encompassing the second and third levels, are pivotal in shaping migrant children’s participation in basic medical insurance. They include the second and third level factors and are also the level that covers the most factors. It includes not only the structural characteristic factors of the education policy (S_2_) of the place of immigration and family’s financial status (S_3_), but also the dynamic characteristic factors of insurance policy publicity (S_8_), including medical security benefit level (S_11_), medical insurance deductible (S_13_), medical insurance reimbursement ratio (S_14_), medical insurance handling service level and efficiency (S_15_) process characteristic factors, and also includes the result characteristic factors of the health level of migrant children (S_16_). These middle-level factors are influenced by deeper factors and, in turn, exert direct or indirect effects on migrant children’s participation in basic medical insurance through shallow inducing factors.

Deep root factors, comprising the fourth and fifth levels, include the household registration system (S_1_), medical insurance system arrangements (S_10_), children’s medication reserve (S_17_) and access of medical services (S_18_). The household registration system (S_1_), as the lowest factor that affects migrant children’s participation in basic medical insurance, has a wide range of influence on other factors. Together with hard factors in the fields of medical insurance, medicine treatment, and medicine, they form the underlying reasons for migrant children to participate in basic medical insurance.

## 6 Discussion

This study applied the DEMATEL-ISM method to systematically analyze the factors influencing migrant children’s participation in basic medical insurance, constructing a multi-layered structure of these factors. The findings reveal that the participation behavior of migrant children is influenced by a complex interplay of various factors, with key determinants centered on the household registration system, the arrangement of medical insurance systems, and the accessibility of medical services.

First, the household registration system has emerged as a crucial factor influencing migrant children’s participation in medical insurance, consistent with findings by Shen Menghan and Wang Zongfan (1, 4). This system plays a decisive role in determining whether migrant children can smoothly enroll in local insurance programs. It not only affects their eligibility but also influences families’ willingness to participate by impacting factors like social integration and children’s access to education. This finding supports calls, such as those by Liu Luchan (6), for reforms that address household registration system-related constraints to reduce barriers to social services for migrants. While previous studies have largely focused on eligibility, this analysis reveals that the household registration system’s broader social and economic implications also shape families’ engagement with medical insurance programs. Therefore, optimizing and reforming the household registration system is essential to removing this barrier.

Second, specific arrangements within the medical insurance system, particularly the ease of cross-regional settlements and the efficiency of administrative procedures, also significantly influence participation. Previous studies, such as those by Yang Lin (7) and Zhang Yongfeng (9), have highlighted the challenges posed by administrative complexities and regional coordination gaps. This study found that the complexity of institutional design and insufficient inter-regional coordination increase the difficulties faced by migrant families during the insurance process. These institutional factors not only affect enrollment rates but also impact the accessibility of medical services for insured children. This finding suggests that simplifying procedural steps and establishing stronger cross-regional coordination mechanisms could enhance participation rates and improve service accessibility, in line with policy recommendations by Li Xuehui and Li Jiaoyuan (8, 22).

Additionally, family economic status and the level of policy awareness play a significant role in determining migrant children’s participation in medical insurance. Consistent with findings by Zhang Yongfeng and Guo Dandan (9, 20), this study reveals that low-income families often view medical insurance as a financial burden rather than a protective benefit. This suggests that increasing financial support, such as subsidies for low-income migrant families, could help reduce participation costs, aligning with recommendations from earlier studies. However, this study uniquely emphasizes the substantial role of policy awareness, showing that many families lack understanding of the benefits and protections offered by medical insurance, which negatively impacts their willingness to participate. Enhancing policy outreach through targeted educational campaigns could effectively improve migrant family engagement with medical insurance, an aspect less emphasized in prior research.

In conclusion, this study has identified the multi-layered and complex interactions among various factors that influence migrant children’s participation in basic medical insurance. Core policy factors interact with economic and social integration factors to collectively shape participation behavior. Future policy efforts should focus on optimizing the household registration system and medical insurance arrangements, while also enhancing policy awareness and economic support measures, in order to improve the coverage rate of migrant children in basic medical insurance.

## 7 Conclusions and suggestions

### 7.1 Conclusion

This study compares with other theories and methodologies, such as Rational Choice Theory, Social Determinants Theory, and Institutional Theory, highlighting the limitations of these approaches in capturing the complex interdependencies among influencing factors. This study addresses these gaps by employing Haken’s synergetics theory, which provides a robust framework for understanding the complex, interdependent relationships among the influencing factors. Drawing on Haken’s synergy theory, this article identifies and analyzes 18 factors influencing migrant children’s engagement with basic medical insurance, examining them through the lens of structure, dynamic, process, and result.

The DEMATEL method is employed to analyze the influencing factors, based on the influencing degree and influenced degree, centrality degree and cause degree ranking to analyze the importance of factors. Additionally, use the ISM method to clarify the logical relationship and action path between each influencing factor. Through the above research path, it was found that migrant children’s participation in basic medical insurance is affected by multiple factors. The 18 influencing factors are interrelated and influence each other, and jointly act on the entire process of migrant children’s participation in basic medical insurance. The integration of the DEMATEL-ISM method allows for both micro and macro-level analysis of the factors, providing a clear visualization of their interrelationships and hierarchical structure. This dual-level analysis is a significant advancement over the single-level analysis typically found in existing studies.

This study has identified several key findings. First, the medical insurance system arrangements (S_10_) hold the highest centrality degree, positioning it at the epicenter of the impact on migrant children’s participation in medical insurance. The household registration system (S_1_) is the causal factor and has the greatest cause degree and the highest influencing degree. Therefore, it also has the greatest influence on other factors. It has strong restriction and driving force and is the most critical factor. Second, the 18 influencing factors are divided into five levels and divided into three tiers: shallow inducing factors, middle influencing factors and deep root factors. The first level is shallow inducing factors and has strong dependence on underlying factors. The second-level and third-level, as middle-level influencing factors, have the ability to transmit influence. The fourth-level and fifth-level are deep root factors and have greater influence. Third, promoting migrant children to participate in basic medical insurance is a systematic project. The interplay and mutual influence among these factors underscore the necessity for key stakeholders: families, schools, the market, society, and government, to provide support and collaborate effectively. This synergy is crucial for ensuring migrant children’s successful inclusion in medical insurance schemes. These findings of this study lead to targeted and effective recommendations for policy interventions. This study provides actionable insights for policymakers to improve the inclusion of migrant children in basic medical insurance.

### 7.2 Policy implications

Deepening the reform of medical insurance introduces new requirements for solving the problem of unbalanced and insufficient medical insurance development, improving the level of medical insurance coordination, enhancing the synergy of the joint reform of medical insurance, medical care, and medicine, and promoting the fairness, inclusiveness, and sustainable development of the medical insurance system. To effectively enhance the participation of migrant children in basic medical insurance, it is essential to implement a prioritized and structured approach. The following recommendations are categorized based on their immediacy and impact, along with detailed operational steps and measures for evaluation.

Immediate actions include reform the household registration system and strengthen coordination and Linkage of medical services.

First, actively promote the reform of the household registration system. The procedure includes the following steps. Firstly, improve the temporary residence permit system and simplify the household transfer procedures for migrant children to facilitate their participation in basic medical insurance at their new place of residence. To facilitate migrant participation in medical insurance in their new place of residence, the government should simplify household registration transfer procedures and reduce the migration threshold. Secondly, implement the point settlement policy to allow migrant workers who have worked and lived in their new place of residence for a long time to accumulate points according to certain standards. After earning points, they can apply to settle in a new place of residence, this will improve the transparency and fairness of the migrant population settlement policy and prevent migrant children from being unable to enjoy basic medical insurance benefits due to household registration issues; simplify household registration transfer procedures. Thirdly, the government should create an inclusive urban culture, improve the city’s tolerance for the migrant population, give full play to the role of labor unions, neighborhood committees, communities and various non-profit organizations, and promote communication between families of migrant children and local residents, as well as interaction between migrant children and locals (37, 38); local governments should combine actual survey data to formulate clear work plans, target the most immediate practical problems of migrant children, and extend urban basic public services and social welfare benefits to migrant children. For example, further building urban schools, lowering the entry threshold for establishing education for migrant children, and continuously improving policies for migrant children to participate in the high school entrance examination and college entrance examination in their place of enrollment, etc.

The evaluation measures include: First, monitor the number of migrant children successfully transferring their household registration. Second, assess the reduction in time and complexity of transfer procedures through surveys and feedback from migrant families. Third, evaluate the level of social integration and satisfaction among migrant children and their families.

Second, strengthen the coordination and linkage of medical care, medical insurance, and medicine to form a joint force to serve migrant children. The procedure includes the following steps. Firstly, further improve medical insurance policies, simplify insurance procedures for migrant children, enhance the cross-regional medical insurance settlement mechanism, increase the convenience of direct settlement for medical treatment in other places, and reduce the economic burden on migrant children families. Secondly, strengthen the construction of grassroots medical institutions, strengthen the allocation of medical resources, and optimize the distribution of medical care resources, improve the quality of medical services, enable migrant children to enjoy high-quality medical services nearby, promote the family doctor system, guide migrant children to rationally use of medical resources, and reduce medical expenses. Thirdly, increase the supply of basic drugs to ensure that migrant children can obtain necessary drugs within the scope of medical insurance. For common and frequently-occurring diseases among migrant children, timely and effective diagnosis and treatment services should be provided to ensure the tangible benefits of medical insurance. At the same time, drug price reform should be promoted through measures such as centralized drug procurement and adjustments to medical insurance drug catalog to control drug prices, ensuring that migrant children can access affordable drug services.

The evaluation measures encompass three aspects. Firstly, track the number of migrant children benefiting from simplified insurance procedures. Secondly, measure improvements in the quality of medical services through patient satisfaction surveys and health outcomes. Thirdly, monitor drug availability and affordability in medical institutions serving migrant populations.

Mid-term actions include improve medical insurance coordination and optimize insurance system design.

First, solidly improve the coordination level of medical insurance. The procedure includes the following steps. Firstly, the government should strengthen the coordination of medical insurance in various regions, improve the convenience of cross-regional medical insurance settlements, reduce the financial burden on migrant children seeking medical treatment elsewhere, and increase their willingness to participate in medical insurance. Secondly, improve and implement medical insurance coordination at the county level. Coordinate at the municipal level and actively explore unified management of the medical insurance system at provincial or national levels, simplify the procedures for migrant children’s participation in medical insurance across different regions, and enhance the efficiency of insurance participation. Thirdly, promote information exchange between medical institutions to facilitate medical treatment for migrant children; establish a dynamic adjustment mechanism to adapt medical insurance policies to the specific needs of migrant children. This mechanism will better meet the medical security needs of migrant children, regularly assess the effectiveness of policy implementation, and adjust medical insurance policies based on the actual circumstances of migrant children.

The evaluation measures encompass three aspects. Firstly, evaluate the efficiency of cross-regional settlements by tracking processing times and patient feedback. Secondly, conduct periodic assessments of the unified management system’s impact on insurance participation rates. Thirdly, measure the effectiveness of information exchange through case studies and data analysis.

Second, optimize the design of the insurance system. The procedure includes the following steps. Firstly, the children’s medical insurance policy should be seamless, provide fully covered, and ensure equal care for every child. To achieve this, it’s urgent to shift from ‘national guidance’ to ‘national leadership’, ensuring children’s medical insurance transcends ‘localization’ and specifically targets migrant children. This involves optimizing the insurance system design to meet the specific needs stemming from their living conditions. Secondly, reform the personal account system and explore the concept of family joint insurance (39); a special fund can be set up to provide medical insurance subsidies for migrant children with financial difficulties and lower their insurance participation threshold; Thirdly, strengthen cooperation with non-governmental organizations to encourage social participation in medical insurance services, aiming to improve the quality and efficiency of services like “Huiminbao” and enhance its health service capabilities; use public spaces like communities and schools to intensify the promotion of medical insurance policies, and popularize medical insurance knowledge through diverse channels, thereby improving the policy awareness of migrant children and their parents, and ensuring a comprehensive understanding of the medical insurance system’s benefits and its protection of individual and family rights and interests (40).

The evaluation measures encompass three aspects. Firstly, monitor the enrollment rates of migrant children in the reformed insurance system. Secondly, assess the financial impact and satisfaction of families participating in joint insurance models. Thirdly, conduct regular evaluations of NGO-led insurance services to ensure quality and efficiency.

The Long-term goals involve establish a unified national health insurance information platform.

The operational steps include: Firstly, develop a national platform for sharing medical insurance data, enabling access to personal information anytime and anywhere. Secondly, implement electronic social security cards for convenient access to medical services. Thirdly, strengthen online medical insurance service platforms for processing insurance, making appointments, and accessing information.

The evaluation measures encompass three aspects. Firstly, measure the usage and accessibility of the national health insurance information platform through user analytics. Secondly, monitor the adoption rate and user satisfaction with electronic social security cards. Thirdly, evaluate the effectiveness of online services through user feedback and service delivery metrics.

### 7.3 Limitations and future research

Despite the comprehensive analysis conducted in this study, certain limitations must be acknowledged. This study primarily focuses on the structural, dynamic, process, and result characteristics, with an emphasis on identifying the logical relationships and hierarchical structure among factors. However, it does not explore the practical effectiveness of specific policy changes across different contexts. Future research could further investigate how these factors perform under varying policy environments and examine the differential impacts that may emerge. The analysis does not account for the potential influence of external macroeconomic factors, such as economic downturns or public health crises, which could significantly affect insurance participation rates. Integrating these macro-level variables into future analyses could yield insights into the resilience and adaptability of migrant children’s participation under varied economic conditions. In addition, although the study offers detailed policy suggestions, the practical implementation of these suggestions may face political, administrative, or logistical challenges not fully explored within the scope of this research. Future studies could focus on the real-world applicability of these recommendations, particularly examining factors influencing the acceptance and effectiveness of policies within migrant communities. Finally, the influence of systemic arrangements, such as the convenience of cross-regional medical service settlements and administrative procedures, on migrant children is complex and multifaceted, warranting further examination and optimization through long-term tracking and field research. Future studies could incorporate behavioral science approaches to explore the acceptance and real-world effectiveness of policy implementation among migrant families, thereby providing support for policy optimization and practical application.

## Data Availability

The original contributions presented in the study are included in the article/supplementary material, further inquiries can be directed to the corresponding authors.

## References

[1] Shen M. Migrant children and their social health insurance participation: evidence from regression discontinuity and difference-in-differences framework. Chin Soc Sec Rev. (2022) 6:74–87.

[2] Yang F. Research on the influence of medical insurance participation behavior andits types on government job satisfaction. Chin J Health Policy. (2021) 14:28–35.

[3] Xu N Gu X Xiang G. Review of Chinese Children’s medical security policy. Health Econ Res. (2020) 37:32–5. doi: 10.14055/j.cnki.33-1056/f.2020.03.006

[4] Wang Z Li J. Current situation, problems and policy suggestions of Children’s medical security in China. Lanzhou Acad J. (2022) 9:113–23.

[5] Zhang Q. A study on family dependence of Chinese children’s medical insurance——take 4 180 samples as an example. Health Econ Res. (2022) 39:11–4. doi: 10.14055/j.cnki.33-1056/f.2022.04.003

[6] Liu L. The dilemma of the cross-provincial medical treatment of migrant population:the origin, policy analysis and removal of institutional barriers. J Sichuan Univ Sci Eng. (2020) 35:31–47.

[7] Yang L Liu J. Obstruction and solutions on the transfer and continuity of medical insurance: a perspective of rural labor employment flows. Nankai J. (2020) 2:69–79.

[8] Li X Xi Z Dong J. The practice and exploration of electronic bill for medical charge. Chin Hosp. (2021) 25:67–9. doi: 10.19660/j.issn.1671-0592.2021.3.21

[9] Zhang Y Chen C. Reevaluation of the implementation effect of China’s social security system. J Xi’an Unive Fin Econ. (2022) 5:1–12. doi: 10.19331/j.cnki.jxufe.2022.05.007

[10] Zhao J Wen X. Effect of basic medical insurance for urban and rural residents on the health of children——an empirical study based on China family panel studies. Soc Sec Stud. (2021) 4:44–56.

[11] Janet C Anna C. Medicaid and child health insurance program improve child health and reduce poverty but face threats. Acad Pediatr. (2021) 21:S146–53. doi: 10.1016/J.ACAP.2021.01.009, PMID: 34740422 PMC9172269

[12] Zhao Z Hu Y. Current status of adolescents’ health insurance participation and its influencing factors in China: evidence based on Chinese education panel survey. Chin J Health Policy. (2019) 12:62–9.

[13] Zhou C Li F. Research on the current situation and influencing factors of migrant children in participating in medical insurance: an example carried out in four cities of southern part of Jiangsu. J Nanjing Med Univ. (2015) 15:91–5.

[14] Bian H Cui J Tang D. The health level of rural left-behind children in underdeveloped areas of China and its management. Soc Sci Res. (2018) 2:114–24.

[15] RLJ T Yang TJ. Addressing population health inequities: investing in the social determinants of health for children and families to advance child health equity. Curr Opin Pediatr. (2022) 35:8–13. doi: 10.1097/MOP.0000000000001189, PMID: 36301135 PMC9803382

[16] Fu X Zhao L Guo W. British pediatric referral pattern and its enlightenment. Chin Health Res. (2017) 20:356–9. doi: 10.13688/j.cnki.chr.2017.16783

[17] Lin J Zhao S. Optimization of children’s basic medical insurance based on flow rate basic tree model. Chin Health Econ. (2022) 41:29–34.

[18] Li Y. Participate in social insurance for Children’s education: impact of the entrance threshold for migrant children on the social insurance participation of floating population. J Financ Econ. (2022) 48:109–23. doi: 10.16538/j.cnki.jfe.20220916.301

[19] Zhuang Q Zhuang L Peng Y. A study on improving the child health security system from the equal health benefits: a case of Beijing. J Bingtuan Med. (2021) 19:8–11.

[20] G D Su Y Guan W. Analysis of medical insurance coverage and its influences on children under five years old: a case study of Wuhan City in Qiaokou. Chin J Health Policy. (2016) 9:61–5.

[21] Du J Zhang H Yu L. Status quo and influencing factors of minority floating population ‘sparticipation in medical insurance. Modern Prev Med. (2020) 47:2881–5.

[22] Li J Fang X. An analysis and reflection on the participation and reimbursement of Children’s social medical Insurance in China: based on the data of China family panel studies. J Jiangxi Univ Fin Econ. (2018) 116:59–68.

[23] Liu W Meng Z Han X. The lmpact of medical insurance on Children’s health. Insur Stud. (2016) 336:77–87. doi: 10.13497/j.cnki.is.2016.04.008

[24] Zhao D Song D Ren J. Analysis on the portability of basic medical Insurance for Migrant Children. Chin Prim Health Care. (2019) 33:5–8.

[25] Chen J Liu K Bu X. Comparison of health service demands and utilization between migrant children and local children in Guangzhou. Chin Gen Pract. (2018) 21:1471–5.

[26] Alphoncina K Amani A Thomas AM. Do household perceptions influence enrolment decisions into community-based health insurance schemes in Tanzania? BMC Health Serv Res. (2021) 21:162–2. doi: 10.1186/S12913-021-06167-Z, PMID: 33607977 PMC7893739

[27] Guo J Dai Y Fu L. Structural equation model of the influencing factors of the medical behavior of floating elderly population. Chin J Health Policy. (2019) 12:35–40.

[28] Chatterji P Ho YC Kamara A Lee J. Trends in common ownership among insurers in Medicare part D. Med Care. (2024) 62:605–11. doi: 10.1097/MLR.0000000000002030, PMID: 38986082 PMC11315633

[29] Ma Y Shi X Sznajder KK Zhao Y Wan Q Chai P. Outpatient depression current care expenditure changes in Liaoning Province from 2015 to 2020: a study based on the “system of health accounts 2011. Front Pharmacol. (2024) 15:151092580. doi: 10.3389/FPHAR.2024.1092580 PMC10839069 38318143

[30] Lin M. System integration, synergetic efficiency in the reform of the social security system: taking the integration of social security in the Yangtze River Delta as an example. Chin Soc Sec Rev. (2022) 6:34–43.

[31] Li B Dong Y. The construction of original innovational dynamic system among enterprises based on the synergy. Sci Manag Sci Technol. (2009) 30:56–60.

[32] Fang Y Li H Zhu M. Influencing factors of the National Coordination of basic pension insurance based on DEMATEL. J Kunming Univ Sci Technol. (2023) 4:1–9. doi: 10.16112/j.cnki.53-1223/n.2023.02.292

[33] Chang J Wang M. Analysis of influencing factors in the pilot process of scientific and technological achievements transformation - based on interpretive structure model. Sci Technol Manag Res. (2017) 37:194–200.

[34] Li Y Yuan Y. Research on the low-carbon transformation mechanism of closed-loop supply chain of manufacturing enterprises under the carbon neutrality goal:based on the DEMATEL-ISM model. Sci Technol Manag Res. (2022) 42:226–34.

[35] Li G Yan Y Liu W. Research on formation factors of miners’ unsafe emotions based on DEMATEL-ISM. Chin Safety Sci J. (2021) 31:30–7. doi: 10.16265/j.cnki.issn1003-3033.2021.07.005

[36] Ni G Li H Cao M. Inducing factors of and intervention countermeasures against unsafe behavior of new generation of construction workers. Safety Environ Eng. (2022) 29:8–16. doi: 10.13578/j.cnki.issn.1671-1556.20210683

[37] Tian X. Implicit Barriers,Urban integration and willingness of agricultural registered migrants to settle down. Issues Agricult Econ. (2022) 516:45–58. doi: 10.13246/j.cnki.iae.2022.12.001

[38] Xiong J. Priority target of Citizenization of migrant workers: policy research based on Citizenization power characteristics and constraints of public investment. Issues Agricult Econ. (2021) 498:60–75. doi: 10.13246/j.cnki.iae.2021.06.007

[39] Li Z Chen C Huang W. Re-Familization: the inevitable choice of basic medical insurance reform. Jinan J. (2023) 45:86–97.

[40] Wang C. Who are the uninsured? An analysis of the population characteristics of China’s urban and rural resident basic medical insurance enrollees. Chin Soc Sec Rev. (2023) 7:76–93.

